# Irritable bowel, chronic widespread pain, chronic fatigue and related syndromes are prevalent and highly overlapping in the general population: DanFunD

**DOI:** 10.1038/s41598-020-60318-6

**Published:** 2020-02-24

**Authors:** Marie Weinreich Petersen, Andreas Schröder, Torben Jørgensen, Eva Ørnbøl, Thomas Meinertz Dantoft, Marie Eliasen, Michael Eriksen Benros, Per Fink

**Affiliations:** 1 The Research Clinic for Functional Disorders and Psychosomatics, Aarhus University Hospital, Aarhus C, Denmark; 2 Center for Clinical Research and Prevention, Bispebjerg and Frederiksberg Hospital, The Capital Region of Denmark, Frederiksberg, Denmark; 3 Department of Public Health, Faculty of Health and Medical Science, University of Copenhagen, Copenhagen C, Denmark; 4 Faculty of Medicine, Aalborg University, Aalborg, Denmark; 5 Mental Health Centre Copenhagen, The Capital Region of Denmark, Copenhagen, Denmark

**Keywords:** Epidemiology, Epidemiology

## Abstract

Prevalence of functional somatic syndromes (FSS) in the general population varies with observed overlap between syndromes. However, studies including a range of FSS are sparse. We investigated prevalence and characteristics of various FSS and the unifying diagnostic construct bodily distress syndrome (BDS), and identified mutual overlap of the FSS and their overlap with BDS. We included a stratified subsample of 1590 adults from a randomly selected Danish general population sample (n = 7493). Telephonic diagnostic interviews performed by three trained physicians were used to identify individuals with FSS and BDS. Prevalence of overall FSS was 9.3%; 3.8% for irritable bowel, 2.2% for chronic widespread pain, 6.1% for chronic fatigue, 1.5% for whiplash associated disorders, and 0.9% for multiple chemical sensitivity. Prevalence of BDS was 10.7% where 2.0% had the multi-organ type. FSS were highly overlapping with low likelihood of having a “pure” type. Diagnostic agreement of FSS and BDS was 92.0%. Multi-syndromatic FSS and multi-organ BDS were associated with female sex, poor health, physical limitations, and comorbidity. FSS are highly prevalent and overlapping, and multi-syndromatic cases are most affected. BDS captured the majority of FSS and may improve clinical management, making the distinction between multi- and mono-syndromatic patients easier.

## Introduction

Functional somatic syndromes (FSS), such as irritable bowel syndrome (IBS), fibromyalgia/chronic widespread pain (FM/CWP), chronic fatigue syndrome (CFS), whiplash associated disorders (WAD), and multiple chemical sensitivity (MCS), are prevalent in various medical settings and cause great impairment in patients and high health care costs for society. A variety of other terms are used in the literature for these syndromes, central sensitization syndromes^1^ and medically unexplained symptoms^2^ among others, which underlines the very different conceptualization of these health conditions in current medicine. However, regardless the clinician’s a-priori conceptualisation, the present lack of objective clinical signs or reproducible paraclinical findings constitutes a diagnostic challenge as the diagnoses in clinical practise rely on subjective symptom reports, exclusion of relevant differential diagnoses, and the medical history^3–9^.

Reviews on IBS, FM, and CFS have shown varying prevalence in general populations: 1–45% for IBS^6,10–13^, 0.5–9% for FM^5,14–16^, and 0.1–2.8% for CFS^17–20^. One population-based study has found a prevalence of WAD of 1.4–2.9%^21^. For MCS, reviews of population-based prevalence also vary tremendously: One review presented prevalence of physician-diagnosed MCS of 0.5% reviews while another have reported a prevalence of MCS of between 1 and 15%^22,23^.

The considerable variation in prevalence may be attributed to the different diagnostic criteria used for each FSS. Furthermore, a substantial overlap of symptoms between various FSS diagnoses has been shown, indicating that the syndromes are not entirely independent conditions but may be different representations of a common phenomenon or belong to a family of related disorders^7,8,24–29^. In order to meet this issue, the unifying diagnostic construct, bodily distress syndrome (BDS) has been proposed^30,31^. It presents with four subtypes of symptom clusters; a cardiopulmonary (CP) type, a gastrointestinal (GI) type, a musculoskeletal (MS) type, and a general symptoms (GS)/fatigue type. BDS can be divided into a single (or oligo)-organ type with symptoms from one or two organ systems and a multi-organ type with symptoms from three or four organ systems. By encompassing a range of FSS, it presents a new way of defining and delimitating these syndromes taking into account the overlaps between FSS^30–32^. The BDS diagnostic construct has recently been verified in a sample from the general Danish population^33^, but until now, only one study about the epidemiology of BDS in a general population-based sample (n = 9656) has been conducted^34^, and further studies on BDS are needed.

FSS and BDS constitute two diagnostic approaches to the umbrella classification functional somatic disorders (FSD) drafted by the European research network EURONET-SOMA^35^, and therefore this term will be used when addressing both FSS and BDS in this paper. As multiple definitions of the various FSS exist, and the choice of criteria for defining individual FSS may therefore be difficult^16,20,36–38^, we will use more broad symptom criteria without symptom overlap in order to gain a genuine picture of the overlap of FSS. Therefore, we use irritable bowel (IB) for describing IBS, CWP to describe FM, and chronic fatigue (CF) for describing CFS.

Many epidemiological studies within FSD use self-reported questionnaires for identification of FSD cases. While this method is cost-effective and appropriate for screening purposes, it only provides a likelihood of having the condition and cannot be used for establishing clinical diagnoses. Instead, a diagnostic interview performed by trained physicians may better delimitate individuals with clinically relevant disorders from individuals with normal bodily reactions and to rule out relevant physical and mental differential diagnoses. This is particularly important within the field of FSD where diagnoses are based on somatic symptom profiles^39^. Using self-reported questionnaires may therefore induce misclassification of both false-positives and false-negatives^40^.

The objectives of this study were to establish prevalence and characteristics of a range of FSDs using two diagnostic approaches: 1) Five FSS (IB, CWP, CF, WAD and MCS) and 2) BDS in the general Danish population using a diagnostic interview performed by trained physicians. Moreover, to study the mutual overlaps of the five FSS and test if the unifying BDS construct succeed in capturing the FSS diagnoses.

## Results

### Study participants

A stratified sample of 2450 (32.7%) participants were invited to participate in the interview, and 1590 (64.9%) accepted and completed. Their median age was 54 years (IQR: 44–63 years), 59.3% were women, and 67.9% had more than three years of vocational training. Comparison analyses revealed no differences between the interviewees and decliners regarding sex and vocational training. However, with a median age of 56 years (IQR: 46–64, p = 0.003), decliners were older than interviewees and had lower risk of a poor self-perceived health (22.7% of interviewees had a poor self-perceived health compared to 16.7% of decliners, RR: 1.4, 95% CI: 1.2–1.6, p < 0.001) and limitations in daily activities (26.2% of interviewees had limitations in daily activities compared to 19.2% of decliners, RR: 1.4, 95% CI: 1.2–1.6, p < 0.001).

For 60 participants, a diagnosis could not be established; either because the participants were under further examination by their family physician or hospital, or because the interviewer could not obtain sufficient information during the interview. In these cases, we conservatively rated the participant as not having an FSD.

### Functional somatic syndromes

Weighted prevalence of having at least one FSS was 9.3% (95% CI: 8.1–10.6), and all FSS were generally more prevalent in women (Table 1). FSS prevalence seemed to be consistent across age groups (Supplementary Material Table 1).

**Table 1:** Prevalence and characteristics of participants with functional somatic syndromes.

	Prevalence of having no FSS N = 1222, 90.7% (95% CI 89.4–91.9)	Prevalence of having one of the below FSS in pure form N = 202, 5.5% (95% CI: 4.5–6.8)					Prevalence of having FSS irrespective of FSS comorbidity N = 357, 9.3% (95% CI 8.1–10.6)							
		IB pure	CWP pure	CF pure	WAD pure	MCS pure	IB	CWP	CF	WAD	MCS	One FSS	Two FSS	≥Three FSS
		N = 70 1.5% (1.1–2.0)	N = 18 0.5% (0.3–0.8)	N = 92 2.7% (2.1–3.6)	N = 10 0.4% (0.2–0.9)	N = 12 0.4% (0.2–0.9)	N = 161 3.8% (3.1–4.6)	N = 96 2.2% (1.8–2.8)	N = 228 6.1% (5.1–7.2)	N = 53 1.5% (1.0–2.0)	N = 33 0.9% (0.6–1.3)	N = 202 5.5% (4.5–6.8)	N = 110 2.7% (2.1–3.5)	N = 45 1.1% (0.8–1.5)
**Basic**														
Age, Median (IQR)	54.0 (44.0–63.0)	**46** (32–60)	55.5 (43–66)	**51** (43.5–58.5)	**44** (33–53)	53.5 (51–58)	**48** (36–58)	52 (45.5–59)	**49** (41–57.5)	50 (42–58)	54 (49–59)	**50** (41–59)	**49** (41–58)	50 (42–57)
Sex; Women vs men, RR*	1 (Ref.)	**1.2** (1.0–1.4)	1.2 (0.9–1.7)	**1.3** (1.1–1.5)	1.0 (0.6–1.7)	**1.7** (1.4–2.0)	**1.4** (1.2–1.5)	**1.4** (1.2–1.6)	**1.4** (1.2–1.5)	**1.3** (1.0–1.5)	**1.4** (1.1–1.7)	**1.3** (1.1–1.4)	**1.4** (1.3–1.6)	**1.4** (1.2–1.7)
Social**														
Cohabiting, RR	1 (Ref.)	0.9 (0.7–1.0)	1.1 (0.9–1.4)	1.0 (0.9–1.1)	1.0 (0.7–1.5)	0.9 (0.6–1.3)	**0.9** (0.8–1.0)	1.0 (0.8–1.1)	0.9 (0.9–1.0)	0.9 (0.8–1.1)	0.9 (0.7–1.1)	1.0 (0.9–1.1)	0.9 (0.8–1.1)	0.9 (0.7–1.1)
Formerly employed, RR	1 (Ref.)	1.0 (0.7–1.4)	1.5 (0.7–3.0)	1.1 (0.8–1.5)	1.1 (0.3–3.3)	1.2 (0.6–2.6)	**1.4** (1.2–1.9)	**2.0** (1.5–2.5)	**1.4** (1.2–1.8)	**1.6** (1.1–2.3)	1.3 (0.8–2.2)	1.1 (0.9–1.3)	**1.6** (1.2–2.1)	**3.0** (2.1–4.1)
Never been employed, RR	1 (Ref.)	0.4 (0.06–2.9)	5.3 (0.9–32.2)	**—**	**—**	**—**	0.9 (0.3–2.5)	**3.6** (1.2–11.0)	1.2 (0.5–3.0)	2.1 (0.3–14.2)	4.5 (0.6–31.3)	0.5 (0.1–2.0)	1.9 (0.8–4.6)	2.4 (0.4–15.8)
Vocational training, OR	1 (Ref.)	1.4 (0.8–2.2)	1.0 (0.4–2.5)	1.1 (0.7–1.7)	0.4 (0.1–1.2)	1.1 (0.4–3.1)	1.1 (0.8–1.5)	1.1 (0.7–1.6)	1.0 (0.8–1.3)	1.1 (0.7–1.8)	1.0 (0.5–2.0)	1.1 (0.8–1.5)	1.0 (0.7–1.4)	1.1 (0.6–1.9)
Physical health**														
Poor self-perceived health^**a**^, RR	1 (Ref.)	1.4 (0.9–2.3)	**2.1** (1.1–4.1)	**2.9** (2.3–3.8)	2.2 (0.9–5.7)	**2.3** (1.2–4.5)	**2.4** (1.9–3.0)	**3.4** (2.8–4.2)	**3.2** (2.7–3.8)	**3.3** (2.5–4.3)	**2.5** (1.7–3.8)	**2.3** (1.8–2.9)	**3.0** (2.3–3.7)	**3.9** (3.1–5.0)
Being limited in daily activities^b^, RR	1 (Ref.)	1.3 (0.9–2.0)	1.7 (0.9–3.3)	**2.3** (1.8–3.0)	1.7 (0.7–4.5)	1.9 (1.0–3.8)	**2.1** (1.7–2.6)	**3.2** (2.6–3.8)	**2.8** (2.4–3.3)	**3.1** (2.4–4.0)	**2.4** (1.7–3.4)	**1.9** (1.5–2.3)	**2.8** (2.3–3.4)	**3.5** (2.8–4.3)
GP diagnosis and comorbidity**														
Having FSS according to a physicianc, RR	1 (Ref.)	**2.9** (2.5–3.5)	**2.0** (1.2–3.4)	**1.3** (1.1–1.5)	**—**	0.7 (0.2–2.4)	**3.0** (2.6–3.5)	**2.7** (2.3–3.2)	**2.3** (2.0–2.7)	**1.6** (1.4–1.8)	**2.2** (1.6–2.9)	**2.1** (1.7–2.5)	**2.6** (2.2–3.2)	**3.1** (2.6–3.7)
Physical comorbidity, RR	1 (Ref.)	**1.5** (1.3–1.8)	**1.3** (1.0–1.6)	1.1 (1.0–1.3)	**—**	**—**	**1.4** (1.3–1.5)	**1.3** (1.2–1.5)	**1.3** (1.2–1.4)	**1.6** (1.4–1.8)	**1.5** (1.2–1.8)	**1.4** (1.3–1.5)	**1.3** (1.2–1.5)	**1.4** (1.2–1.6)
Mental comorbidity, RR	1 (Ref.)	1.3 (0.6–2.7)	1.7 (0.4–6.3)	**2.9** (1.9–4.6)	**—**	1.2 (0.1–8.2)	**2.4** (1.6–3.6)	**3.2** (2.1–4.9)	**3.3** (2.4–4.6)	1.0 (0.4–2.7)	1.8 (0.7–4.5)	**1.9** (1.3–2.9)	**3.0** (2.0–4.6)	**3.4** (1.9–5.9)

Abbreviations: FSS = functional somatic syndrome; IB** =** irritable bowel; CWP** =** chronic widespread pain; CF** =** chronic fatigue; WAD** =** whiplash associated disorders; MCS** =** multiple chemical sensitivity.

IQR = interquartile range; RR = relative risk; OR = odds ratio.

*Adjusted for age.

**Adjusted for age and sex.

^a^Fair or poor health^89^.

^b^All of/most of/some of the time^89^.

^c^Received a diagnosis of at least one of the following: Irritable bowel syndrome, fibromyalgia, chronic fatigue syndrome, whiplash associated disorders, multiple chemical sensitivity by a general practitioner.

**Bold:** Significantly different from participants not having any FSS (P < 0.05). “-“could not be estimated because of too few observations.

Prevalence of cases with only one FSS, i.e. having a “pure” type of FSS, was 5.5% (95% CI: 4.5–6.8) (Table 1). These cases tended to be younger than non-cases, and they had higher risk of having a poor health and physical and mental comorbidities. However, these associations were only statistically significant for some of the pure FSS (Table 1).

**Figure 1:**
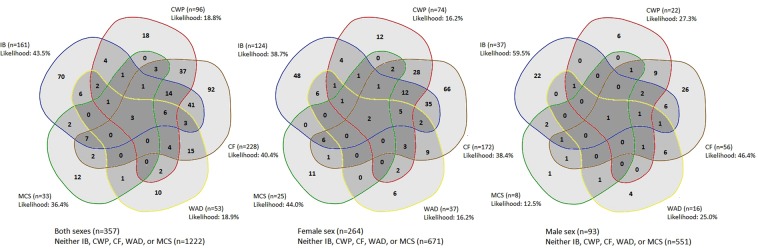
Overlap of functional somatic syndromes.

A substantial fraction of cases within each FSS type had more than one FSS, and 20 out of 26 possible combinations of the five FSS were found. The likelihood of having only one FSS in its pure form was low ranging from 18.8% for CWP to 43.5% for IB (Fig. 1).

Compared with non-cases, multi-syndromatic FSS cases were generally younger and they had higher risk of being unemployed, to have a poor self-perceived health, to have limitations in daily activities, to have physical and mental comorbidities, and to have received a FSS diagnosis by a physician (Table 1). In contrast to cases with only one FSS, the majority of these associations were strong and statistically significant. Hence, multi-syndromatic FSS cases seemed to be stronger associated with several negative outcomes than those only having one FSS. Data on age, sex, social factors, physical health, and comorbidity are displayed as percentages in Supplementary Material Table 2.

### Bodily distress syndrome

BDS had a weighted prevalence of 10.5% (95% CI: 9.3–11.9) (Table 2). As for FSS, prevalence of BDS was also consistent across age groups (Supplementary Material Table 1).

**Table 2:** Prevalence and characteristics of participants with and without bodily distress syndrome.

	No BDS	Single-organ BDS	CP subtype	GI subtype	MS subtype	GS subtype	Multi-organ BDS
	N = 1177 89.5% (88.1–90.7)	N = 327 8.5% (7.3–9.7)	N = 47 1.3% (0.9–1.9)	N = 173 4.0% (3.3–4.8)	N = 107 3.0% (2.3–3.8)	N = 111 3.1% (2.4–4.0)	N = 86 2.0% (1.6–2.6)
**Basic**							
Age, Median (IQR)	55.0 (45.0–64.0)	**52.0** (42.0–60.0)	**51.0** (37.0–59.0)	**53.0** (42.0–61.1)	**53.0** (47.0–60.0)	**49.0** (40.0–56.0)	**48.5** (39.0–57.0)
Sex; Women vs men, RR*	1 (Ref.)	**1.3** (1.2–1.4)	**1.2** (1.0–1.5)	**1.3** (1.1–1.4)	**1.4** (1.2–1.5)	**1.3** (1.2–1.5)	**1.5** (1.3–1.7)
Social**							
Cohabiting, RR	1 (Ref.)	**0.9** (0.8–1.0)	0.9 (0.7–1.1)	**0.9** (0.8–1.0)	0.9 (0.8–1.0)	**0.9** (0.7–1.0)	0.9 (0.7–1.0)
Formerly employed, RR	1 (Ref.)	**1.3** (1.1–1.6)	1.4 (0.9–2.2)	**1.3** (1.0–1.6)	**1.7** (1.3–2.3)	**1.5** (1.2–2.1)	**1.6** (1.2–2.2)
Never been employed, RR	1 (Ref.)	0.8 (0.3–2.0)	0.7 (0.1–4.8)	0.4 (0.1–1.8)	2.6 (0.8–8.1)	0.6 (0.2–3.0)	**2.5** (1.1–6.1)
Vocational training, OR	1 (Ref.)	1.0 (0.8–1.3)	1.2 (0.7–2.1)	1.1 (0.8–1.5)	0.9 (0.6–1.3)	1.0 (0.7–1.5)	1.0 (0.7–1.6)
Physical health**							
Poor self-perceived health^a^, RR	1 (Ref.)	**2.3** (1.9–2.8)	**2.2** (1.5–3.4)	**2.0** (1.5–2.6)	**2.7** (2.1–3.5)	**3.0** (2.4–3.8)	**3.8** (3.0–4.8)
Being limited in daily activities^b^, RR	1 (Ref.)	**2.1** (1.8–2.5)	**2.1** (1.5–3.1)	**1.6** (1.3–2.1)	**2.6** (2.1–3.3)	**2.7** (2.1–3.3)	**2.9** (2.3–3.5)
GP diagnosis and comorbidity**							
Having FSS according to a physician^c^, RR	1 (Ref.)	**2.3** (1.9–2.6)	**2.0** (1.4–2.9)	**2.7** (2.3–3.2)	**2.2** (1.8–2.7)	**1.9** (1.5–2.5)	**2.6** (2.1–3.2)
Physical comorbidity, RR	1 (Ref.)	**1.3** (1.3–1.5)	**1.4** (1.2–1.7)	**1.4** (1.3–1.5)	**1.2** (1.1–1.4)	**1.3** (1.2–1.5)	**1.3** (1.1–1.5)
Mental comorbidity, RR	1 (Ref.)	**2.8** (2.0–4.0)	**3.1** (1.7–5.8)	**2.7** (1.8–4.1)	**2.5** (1.5–4.2)	**3.9** (2.6–5.9)	**4.3** (2.7–6.6)

Abbreviations: BDS = bodily distress syndrome; CP = cardiopulmonary; GI = gastrointestinal; MS = musculoskeletal; GS = general symptoms type.

IQR = interquartile range; RR = relative risk; OR = odds ratio.

*Adjusted for age.

**Adjusted for age and sex.

^a^Fair or poor health^89^.

^b^All of/most of/some of the time^89^.

^c^Received a diagnosis of at least one of the following: Irritable bowel syndrome, fibromyalgia, chronic fatigue syndrome, whiplash associated disorders, and multiple chemical sensitivity by a general practitioner.

**Bold:** Significantly different from participants not having BDS (P < 0.05).

Cases of BDS were younger than non-cases, and they had high risk of being unemployed, having a poor health, limitations in daily activities, physical and mental comorbidity, and to have received a FSS diagnosis by a physician (Table 2). All associations were especially strong for the multi-organ type of BDS. Data on age, sex, social factors, physical health, and comorbidity are displayed as percentages in Supplementary Material Table 3.

### Overlaps of functional somatic syndromes and bodily distress syndrome

The prevalence of those having at least one FSS and/or BDS was 12.1% (95% CI: 10.7–13.6). Overall diagnostic agreement between any of the five FSS and BDS was 92.0% (95% CI: 90.6–93.3) (Table 3), corresponding to a kappa of 0.78, p < 0.0001. Compared to FSS, BDS had a sensitivity of 90.2% (95% CI: 86.6–93.1) and a specificity of 92.6% (95% CI: 90.9–94.0). A Receiver Operating Characteristics curve is displayed in Supplementary Material Fig. 1.

**Table 3:** Diagnostic agreement between functional somatic syndromes and bodily distress syndrome.

		*Diagnostic (observed) agreement; % (95% CI)*					
		At least one FSS	IB	CWP	CF	WAD	MCS
		(n = 357)	(n = 161)	(n = 96)	(n = 228)	(n = 53)	(n = 33)
BDS	(n = 413)	92.0 (90.6–93.3)	83.5 (81.6–85.3)	79.9 (77.9–81.9)	86.1 (84.3–87.8)	76.1 (74.0–78.2)	75.2 (73.0–77.3)
Multi-organ BDS	(n = 86)	82.7 (80.8–84.6)	91.2 (89.7–92.6)	94.0 (92.7–95.1)	90.1 (88.5–92.5)	93.4 (92.0–94.6)	94.3 (93.0–95.4)
Single-organ BDS	(n = 327)	86.7 (84.9–88.3)	82.1(80.1–84.0)	79.9 (77.9–81.9)	81.7 (79.7–83.6)	79.4 (77.3–81.4)	78.9 (76.8–80.9)
*CP subtype*	*(n* = *47)*	*78.0 (75.8–80.0)*	*88.3 (86.6–89.8)*	*91.1 (89.5–92.4)*	*85.1 (83.2–86.8)*	*93.9 (92.7–95.1)*	*95.5 (94.3–96.4)*
*GI subtype*	*(n* = *173)*	*86.7 (84.9–88.4)*	*94.6 (93.3–95.7)*	*86.2 (84.3–87.9)*	*83.9 (81.9–85.7)*	*87.2 (85.4–88.9)*	*88.0 (86.3–89.6)*
*MS subtype*	*(n* = *107)*	*84.1 (82.1–85.9)*	*87.0 (85.2–88.7)*	*96.3 (95.2–97.2)*	*88.1 (86.4–89.7)*	*91.8 (90.3–93.1)*	*92.1 (90.6–93.4)*
*GS subtype*	*(n* = *111)*	*87.6 (85.8–89.2)*	*87.2 (85.4–88.9)*	*91.1 (89.6–92.5)*	*95.4 (94.2–96.4)*	*92.4 (90.9–96.7)*	*91.6 (90.1–93.0)*

Abbreviations: FSS = functional somatic syndrome; IB = irritable bowel; CWP = chronic widespread pain; CF = chronic fatigue; WAD = whiplash associated disorders; MCS = multiple chemical sensitivity; BDS = bodily distress syndrome; CP = cardiopulmonary; GI = gastrointestinal; MS = musculoskeletal; GS = general symptoms type; CI = confidence interval.

## Discussion

To our knowledge, this is the first study to simultaneously investigate prevalence and characteristics of the three most investigated FSS (IB, CWP, CF) and two exposure-defined FSS (MCS, WAD) in the general population by means of a clinical diagnostic research interview performed by trained family physicians over the telephone. Moreover, we also assessed a new unifying diagnostic construct, BDS, and its ability to capture the more established diagnoses. Overall weighted prevalence of having at least one FSS was 9.3%, and while most of the overall case group had only one FSS, the majority within each FSS type had FSS comorbidity. Overall weighted prevalence of BDS was 10.5% (95% CI: 9.3–11.9), and this constituted mostly single-organ BDS, i.e. patients with symptoms from one or two organ systems. Compared to non-cases, multi-syndromic FSS and multi-organ BDS were strongly associated with unemployment, poor health, limitations in daily activities, and physical and mental comorbidity. These associations were less strong for those only having one FSS or single-organ BDS indicating that individuals with multiple FSD are more burdened. Overall diagnostic agreement between FSS and BDS was high (92.0%, kappa 0.78), sensitivity and specificity of BDS were high (>90%), and the BDS construct captured the majority of FSS categories.

Other population-based studies including several FSS simultaneously have reported prevalence of IBS ranging from 3.5% to 12.6%, prevalence of FM ranging from 1.9% to 9.4%, and prevalence of CFS/CF ranging from 0.8% to 12.6%^34,41–44^. Hence, our results on prevalence of IB, CWP, and CF are in line with these studies, even though we found lower prevalence of IB and CWP. One reason for this could be that all mentioned studies have used questionnaires as method for case identification which could cause some false positive cases overestimating the various FSS prevalence.

To our knowledge, interviews have been used in population-based studies to study somatic symptoms^45,46^ and in more selected patient samples to study prevalence of FSS and BDS^30,46–48^, but until now, no other population-based studies on interview-based FSS or BDS prevalence exist.

In a recent DanFunD study, we reported a questionnaire-based prevalence of having at least one FSS and/or BDS that was almost 50% higher than in the current study^34^. This difference may be explained by the fact that in the current study, we did not include participants with conventionally-defined disease that could account for the physical symptoms as FSD cases.

Our results on case characteristics are in line with several other studies on specific FSS

It is a consistent finding that the female sex dominates in both IBS^10–13,49^, FM^5,14,16,50^, CFS^17,19,51^, WAD^52^, and MCS^22,23^. However, one study did not find any pronounced predominance of the female sex in FM^53^. In that study, FM prevalence and sex ratio were estimated using the 2016 Fibromyalgia criteria in one sample of patients diagnosed with FM but no other rheumatoid or inflammatory diseases and one sample of patients diagnosed with rheumatoid arthritis but no FM. Since the current study includes individuals sampled from the general population, it may to a lesser extent have such risk of selection bias.

Unemployment and the inability to maintain a job have also previously shown to be associated with both IBS^10,11^, FM^16,50^, and CFS^17,51^.

In the current study, all FSS are highly associated with self-rated poor quality of life, i.e. having a poor health or limitations in daily activities, which is similar to findings in other studies on IBS^6,10–13,49^, FM^16,50^, CFS^17,51^, WAD^52,54,55^, and MCS^23^.

Other studies also found high comorbidity with other diseases in all FSS. This counts for both physical^5,6,14,18,22,50,52,54,56,57^ and mental disease^5,10,14,17–19,21–23,50–52,54,58,59^ as well as other FSS^6,17,21,29,51,54,56,58,59^.

Studies on general populations including a range of FSS have shown the same associations^34,41–45,60^, and as in the current study, mental comorbidity seemed pronounced in CFS^34,43^.

Only few studies have been made on BDS, and these constitute mainly studies on clinical samples. These studies also find cases of BDS being associated with the female sex^34,61,62^, unemployment^62–64^, poor health, limitations in daily functioning^62–65^, high comorbidity with physical disease and other FSS, and mental disorders^34,62,64,65^.

The debate on how to understand, define, and delimitate FSD has been going on for decades^7,8,24,26,27,66^. It has divided debaters into splitters, defining the disorders as distinct entities, and lumpers arguing that all FSD are manifestations of the same underlying disorder^26,67,68^.

We found a low likelihood of having the “pure” type of each of the investigated disorders thus questioning the current clinical practice that focuses on one disorder at a time. Even more importantly, our results question the usability of specific guidelines for single (i.e. “pure”) syndromes as most patients presenting at the clinic have FSS comorbidities. Currently, even though it has been widely recognized in research that the FSD are not discrete diseases, patients are often managed based on this perspective when attending the health care system.

Our results indicate that the vast majority of e.g. patients with IBS/IB in gastroenterological departments also have at least one other FSS, for which they are most likely not being treated in a specialized setting^69^.

Current clinical practice bears the risk of neglecting those multi-syndromatic patients. Especially the shift of main symptoms or syndromes over time is quite often overlooked, as each FSS is assessed and treated as a distinct condition. In the current study, the group of multi-syndromatic individuals constituted 20 out of 26 possible combinations of the five investigated FSS, comprising 43.4% of all individuals with FSS in the general population. Giving each combination of disorders a specific assessment and treatment plan in the clinic would not be feasible. Hence, the importance of giving each patient an assessment, acknowledging all presenting symptoms, should be emphasized^9,70–72^.

The BDS diagnostic construct may provide a solution for this defining the FSD as a common phenomenon but with distinct subtypes^30^. The diagnostic agreement between FSS and BDS was high which has also been shown in studies on more selected patient samples^30,32^. Our results underline previous studies showing that the BDS concept can easily distinguish between multi-syndromic patients and those with symptoms from only one or two organ systems. This distinction may improve clinical management, and BDS shows a promising compromise between the splitters’ and lumpers’ perspectives in managing patients with FSD and in future research^26,67^. This suggested unifying phenotype construct based on empirical data may suggest a common underlying etiology of the FSD. Most studies into the genetic and environmental risk factors for different FSS have shown that the co-occurrence of FSS may be caused by shared genetics and both common (e.g. a trauma) but also specific (e.g. infections) environmental risk factors^28,29,73,74^. However, another study suggests that CWP may mainly be genetically associated with fatigue^59^.

Whether or not these discrepancies could be attributable to methodological differences between studies is unclear. A main problem with studies on the various FSS is that most have focused on only one or a few syndromes at a time, viewing the FSS as distinct syndromes without taking FSS comorbidity into account. In future research, approaching the FSD with criteria acknowledging them as being both mono- and multi-syndromatic conditions may lay down the avenue for better studies on their etiology.

This study has several strengths. First, the large sample of participants completing the interviews is a substantial strength in the study adding power to the statistical analyses. Second, the use of trained physicians and semi-structured diagnostic research interviews provides a better possibility for distinguishing between functional and differential diagnoses than questionnaires, self-reported diagnoses, or layman implemented interviews. The experienced clinicians have the capacity to judge clinical relevance and complexity of each symptom and assess if the symptom clusters could be a manifestation of a FSD. Hence, the case assignment was based on specialized, medical knowledge. Third, we assessed the FSD according to two diagnostic approaches: Five different FSS with distinct criteria without mutual overlap of symptoms and the unifying concept of BDS.

However, the study also has some limitations. First, the diagnostic interviews were conducted by telephone. Thus, we merely relied on information from the participants and did not have access to medical records or the possibility of conducting further medical tests or physical clinical examinations, which is required to make an exhaustive, clinically valid diagnosis. Second, different traditions within each medical speciality together with the absence of a gold standard for diagnosing FSD might cause some cases to be falsely labelled as non-cases due to multi-morbidity, i.e. where conventionally defined physical disease was present together with the FSD. Furthermore, we cannot be completely certain of the RIFD’s ability to diagnose at a detailed level, e.g. to detect single subtypes of FSS and BDS. Third, using others of the multiple prevailing criteria for defining the various FSS may have provided us with different results, but we preferred to include criteria commonly used in epidemiological research. Furthermore, the included criteria did not have symptom overlap which is a problem of many of the specialty specific definitions developed in highly selected patient samples. Moreover, the included less restrictive criteria may better capture the broader spectrum of FSS that may be expected in a general population sample. Finally, only 64.9% accepted the interview invitation, and the majority (98.3%) of those who declined was high scores in the symptom questionnaires. Loss of participants to the second study phase is a general limitation of the two-phase design^75^, and it might have influenced the found associations concerning characteristics of the cases and non-cases. However, as the results from this study are in line with results in other studies on the complete DanFunD cohort^34,76^, we believe our results to be reliable.

## Conclusions and Perspectives

Regardless of diagnostic criteria, FSD are highly prevalent and overlapping. The impact on functioning and quality of life is considerable with strong associations with poor health, limitations in daily activities, and physical and mental comorbidity. Some pure FSS categories seem more burdensome, but cases with multiple FSS or multi-organ BDS are most affected. While the distinct FSS diagnoses may have some merits in specific clinical settings, the unifying BDS construct makes it easy to distinguish between multi-syndromic patients, who are currently in risk of being neglected, and those with symptoms from only one or two organ systems. This distinction may improve current clinical management of FSD.

## Methods

### Study participants and data collection

**Figure 2:**
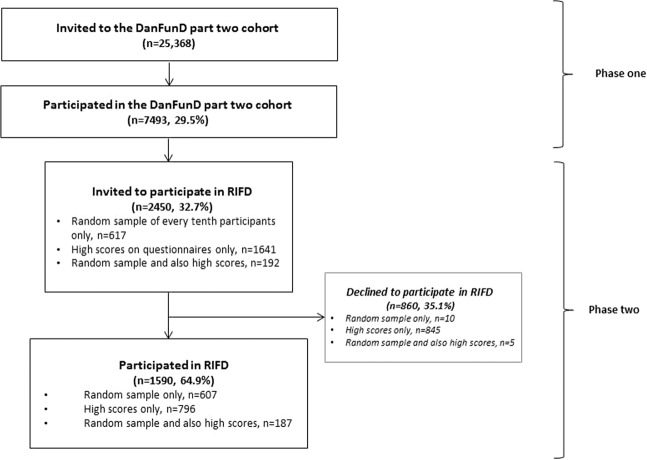
Flow of study participants.

This study was designed as a cross-sectional two-phase study (Fig. 2) using data from the Danish Study of Functional Disorders (DanFunD) part two cohort^77^. In brief, the cohort was established from 2012 to 2015 with the purpose of investigating FSD in the general Danish population. DanFunD part two consists of 7493 (29.5%) adults sampled from the general Danish population and randomly obtained from the nationwide Danish Civil Registration System. In phase one, participants filled in symptom questionnaires for physical symptoms specific for FSS and BDS, social factors, and overall health, among others. Prevalence of FSS and BDS obtained from these questionnaires has been reported elsewhere^34^. In phase two, the questionnaires formed the basis for identifying a stratified subsample invited to participate in the Research Interview for Functional somatic Disorders (RIFD)^78^, derived from a modified version of the comprehensive psychiatric interview Schedules of Clinical Assessment in Neuropsychiatry (SCAN)^79^. The RIFD interview assesses symptoms for a range of FSD; IBS/IB, FM/CWP, CFS/CF, WAD, MCS, and BDS, and it has shown to be a feasible instrument for use in large epidemiological studies for identifying individuals with FSD. In a small preliminary study, RIFD showed acceptable criterion validity and interrater reliability^78^.

The subsample was combined by a random sample of one tenth of all DanFunD part two cohort participants and participants with high scores on the BDS checklist^80^ or Whiteley-7 scale^81^ in the symptom questionnaires. For being a high score on the BDS checklist, participants had to be bothered “quite a bit” or “a lot” from symptoms from at least one symptom cluster and report additional impairment on ≥6 on a continuous 0–10 scale. For being a high score on the Whiteley-7 scale, participants had to be bothered “quite a bit” or “a lot” from symptoms and report additional impairment on ≥6 on a continuous 0–10 scale. All interviews were conducted by telephone by three trained family physicians. Detailed information on the general procedure of RIFD has been described in detail elsewhere^78^.

Written informed consent was obtained from each participant before participation.

The study was approved by the independent ethics committees the Ethical Committee of Copenhagen County (Ethics Committee: KA-2006-0011; H-3-2011-081; H-3-2012-0015) and the Danish Data Protection Agency.

All methods were carried out in accordance with the relevant guidelines and regulations.

### Measurements

#### Diagnostic criteria

Post-hoc diagnostic criteria were used to assign case status on the basis of the obtained symptoms from the interviews (Table 4). The diagnostic criteria by Kay *et al*.^82^ for IBS/IB, the diagnostic criteria by White *et al*.^83^ (modified from the American College of Rheumatology 1990 FM criteria^84^) for FM/CWP, the diagnostic criteria by Chalder *et al*.^85^ for CFS-like symptoms/CF, and the diagnostic criteria by Fink *et al*.^61^ for BDS. Diagnostic criteria for WAD were defined as having impairing neck pain that could be attributed to an accident^86^. Diagnostic criteria for MCS were defined as having impairing symptoms believed to be attributed to exposure to fragrances or chemical substances^87,88^.

**Table 4:** Post-hoc diagnostic criteria of functional somatic syndromes and bodily distress syndrome.

Syndrome	Diagnostic criteria
Irritable bowel^82^	Participants with 1) *abdominal pain* and 2) *distension* and, in addition, either 3) *borborygmi* or 4) *altering stool consistency*, or both.
Chronic widespread pain^83^	Participants with 1) *pain in muscles, bones, or joints*; and 2) *pain in shoulders, arm, or hands*; and 3) *pain in legs or feet*; and 4) *pain in neck, chest, or back*.
Chronic fatigue^85^	Participants with ≥4 of the following: 1) *Tiredness*; 2) *the need to rest more*; 3) *sleepiness or drowsiness*; 4) *problems starting things*; 5) *lack of energy*; 6) *less muscle strength*; 7) *weakness*; 8) *difficulties concentrating*; 9) *making slips of the tongue when speaking*; 10) *difficulties saying the right words*; and 11) *memory problems*.
Whiplash associated disorders^86^	Participants stating to be bothered by impairing *neck pain* following an *accident*.
Multiple chemical sensitivity^87,88^	Participants stating to have been exposed by at least two *unrelated exposures* and to have been bothered by symptoms from at least *two organ systems*; at least one being the central nervous system. The symptoms should have *significant impact* on life style together with *social or occupational limitation*.
Bodily distress syndrome^61^	Participants with ≥3 symptoms from different symptom clusters together with impairment; 1) *a cardiopulmonary cluster*, 2) *a gastrointestinal cluster*, 3) *a musculoskeletal cluster*, 4) *a general symptom cluster*.
	Participants having 1 or 2 symptom clusters or at least 4 symptoms across all symptom clusters single-organ bodily distress syndrome. Participants having ≥3 symptom clusters had multi-organ bodily distress syndrome.

#### Characteristics

Cases and non-cases were described and compared by self-reported measures from the questionnaires: Cohabitation, employment, vocational training, self-perceived health, limitation in daily activities, and whether the participant had received a diagnosis of one of the five FSS by a physician. Self-perceived health and limitations in daily activities were measured with two single questions from the 12-item Short Form Health Survey (SF-12)^89^. Self-perceived health was measured on a five-point Likert scale from “excellent” to “poor”, and answers were dichotomized into “poor self-perceived health” (fair/poor) and “good self-perceived health” (excellent/very good/good). Limitations in daily activities were measured on a five-point Likert scale (“all the time” to “none of the time”) dichotomized into “limited in daily activities” (all of the time/most of the time/some of the time) and “not limited in daily activities” (a little of the time/none of the time). The interviewer assessed physical comorbidity based on whether or not the participant had received diagnoses of any of the following pre-defined diseases: Diabetes, asthma, joint disease, cardiac disease, hypertension, and pulmonary disease. Furthermore, it was possible for the interviewer to register if the participant had another disease which was not included in the pre-defined list of diseases. Mental comorbidity was also assessed in the interview using post-hoc ICD-10 criteria to define any state of major depressive disorder and single items assessing anxiety disorders (panic attacks, social phobia, and generalized anxiety)^78^.

### Data analysis

Data were analysed using Stata 15.0 for Windows (StataCorp LLC, College Station, USA).

Prevalence of FSS and BDS was calculated with 95% confidence intervals using weighted logistic regression with the interviewed sample fraction as sample weights to correct for the skewness introduced by the stratified sampling procedure. With information on the interview sampling mechanism (randomly selected, selected as high score, randomly selected but also with high score) together with information on age and sex, assignment of sample weights of each of the participants was made. This approach gave information on how many participants from the screening questionnaire sample that were represented by each of the interviewed participants, generalizing the prevalence to the whole DanFunD part two sample of 7493 individuals^75^.

Difference in age between cases and non-cases were compared with a Wilcoxon-Mann-Whitney test for non-normally distributed data. Sex, cohabitation, employment, self-perceived health, self-perceived limitations in daily activities, information on obtained FSS diagnoses, and comorbidity were presented as dichotomous outcomes with risk ratios calculated with general linear models with binomial family and log link. Vocational training was presented as an ordered categorical outcome with odds ratios calculated with ordered logistic regression. Sex was adjusted for age, while social parameters, physical health, and comorbid disease were adjusted for age and sex.

Interviewed participants and participants declining to participate in the RIFD were compared on age, sex, vocational training, self-perceived health, and limitations in daily activities.

Mutual overlaps of the various FSS were estimated and shown in Venn-diagrams using the web-tool Venn diagram maker from the website Bioinformatics & Evolutionary Genomics^90^.

Diagnostic agreement between FSS and BDS was evaluated with Cohen’s Kappa, and sensitivity and specificity of BDS were calculated.

## Supplementary information

Supplementary information.

## Data Availability

The datasets generated during and/or analysed during the current study are available on reasonable request from the DanFunD project leader Thomas Dantoft by email: Thomas.Meinertz.Dantoft@regionh.dk.
