# Supplementary material for: Irritable bowel, chronic widespread pain, chronic fatigue and related syndromes are prevalent and highly overlapping in the general population: DanFunD
Source: Sci Rep. 2020 Feb 24;10:3273. doi: 10.1038/s41598-020-60318-6 (PMC7039919; doi:10.1038/s41598-020-60318-6)
Supplement: Supplementary file 1 — Supplementary information. [file 41598_2020_60318_MOESM1_ESM.pdf]

Supplementary information for the paper:

"Irritable bowel, chronic widespread pain, chronic fatigue and related syndromes are prevalent and highly overlapping in the general population: DanFunD"

Authors:

Marie Weinreich Petersen, Andreas Schröder, Torben Jørgensen, Eva Ørnbøl, Thomas Meinertz Dantoft, Marie Eliasen, Michael Eriksen Benros, Per Fink

Table 1: Weighted prevalence of functional somatic syndromes and bodily distress syndrome across sex and age groups

	**Women, age (years); % (95% CI)**					**Men, age (years); % (95% CI)**				
	All (n=942)	18-39 (n=165)	40-49 (n=231)	50-59 (n=271)	60-76 (n=275)	All (n=648)	18-39 (n=109)	40-49 (n=111)	50-59 (n=174)	60-76 (n=254))
**FSS**	**11.0** (9.4-12.8)	**14.4** (10.4-19.5)	**13.5** (10.1-17.8)	**11.6** (8.8-15.2)	**7.2** (5.0-10.2)	**7.2** (5.6-9.4)	**8.6** (4.6-15.3)	**12.8** (7.7-20.5)	**6.9** (4.1-11.3)	**4.0** (2.6-6.3)
**IB**	**4.9** (4.0-6.2)	**8.8** (5.8-13.1)	**5.5** (3.6-8.5)	**4.5** (2.8-7.1)	**2.9** (1.9-4.5)	**2.4** (1.6-3.7)	**2.7** (1.0-7.1)	**5.6** (2.6-11.6)	**1.7** (0.6-4.3)	**1.3** (0.6-2.5)
IB pure	*1.7 (1.2-2.5)*	*3.0 (1.7-5.4)*	*1.9 (1.0-3.4)*	*1.3 (0.4-3.5)*	*1.3 (0.7-2.4)*	*1.2 (0.7-2.2)*	*2.3 (0.8-6.8)*	*2.1 (0.7-6.6)*	*0.6 (0.2-2.1)*	*0.7 (0.3-1.6)*
**CWP**	**2.8** (2.1-3.7)	**2.4** (1.3-4.4)	**3.7** (2.2-6.1)	**3.0** (1.9-4.7)	**2.4** (1.3-4.2)	**1.6** (0.9-2.6)	**0.6** (0.1-2.6)	**2.4** (0.8-7.0)	**2.3** (1.1-5.0)	**1.0** (0.5-2.3)
CWP pure	*0.6 (0.3-1.3)*	*0.4 (0.1-2.0)*	*0.8 (0.2-3.8)*	*0.1 (0.03-0.6)*	*0.9 (0.3-2.8)*	*0.3 (0.1-0.8)*	*0.1 (0.02-1.0)*	*0.4 (0.05-2.5)*	*0.4 (0.1-1.9)*	*0.4 (0.1-1.4)*
**CF**	**7.4** (6.1-8.9)	**10.9** (7.3-15.8)	**9.8** (6.7-13.7)	**8.3** (5.9-11.5)	**3.3** (2.0-5.6)	**4.6** (3.2-6.4)	**3.7** (1.5-9.0)	**9.1** (4.9-16.3)	**4.6** (2.4-8.5)	**2.6** (1.4-4.6)
CF pure	*2.9 (2.1-4.0)*	*3.8 (1.9-7.2)*	*4.0 (2.2-7.2)*	*3.0 (1.7-5.2)*	*1.7 (0.8-4.0)*	*2.5 (1.5-4.1)*	*2.2 (0.5-8.3)*	*4.2 (1.7-10.2)*	*3.2 (1.3-7.3)*	*1.3 (0.5-3.2)*
**WAD**	**1.5** (1.0-2.2)	**1.0** (0.4-2.5)	**1.7** (0.9-3.1)	**2.8** (1.6-5.0)	**0.6** (0.2-1.5)	**1.4** (0.8-2.6)	**3.1** (1.0-8.9)	**2.0** (0.6-6.6)	**1.2** (0.4-3.9)	**0.5** (0.1-1.5)
WAD pure	*0.2 (0.1-0.5)*	*0.1 (0.02-0.9)*	*0.2 (0.05-0.8)*	*0.5 (0.1-1.5)*	*0 (-)*	*0.6 (0.2-1.8)*	*2.3 (0.6-8.9)*	*1.2 (0.2-6.9)*	*0 (-)*	*0 (-)*
**MCS**	**1.1** (0.7-1.9)	**0.7** (0.2-1.9)	**0.6** (0.2-1.7)	**1.6** (0.9-3.0)	**1.3** (0.4-3.8)	**0.5** (0.2-1.3)	**0.4** (0.1-2.9)	**0.2** (0.1-0.9)	**1.0** (0.2-4.1)	**0.4** (0.1-1.4)
MCS pure	*0.6 (0.3-1.4)*	*0 (-)*	*0.3 (0.1-1.3)*	*0.7 (0.3 -1.7)*	*1.0 (0.2-3.8)*	0.2 (0.03-1.3)	*0 (-)*	*0 (-)*	*0.7 (0.1-4.5)*	*0 (-)*
**1 FSS**	**6.0** (4.8-7.4)	**7.3** (4.7-11.0)	**7.3** (4.8-10.8)	**5.5** (3.7-8.2)	**5.0** (3.1-7.9)	**4.8** (3.5-6.7)	**7.0** (3.4-13.9)	**8.6** (4.3-17.1)	**4.8** (2.5-9.1)	**2.3** (1.2-4.3)
**2 FSS**	**3.7** (2.7-4.7)	**5.4** (3.0-9.6)	**4.9** (3.1-7.8)	**4.3** (2.6-6.9)	**1.1** (0.6-2.2)	**1.8** (1.1-2.9)	**1.2** (0.4-3.6)	**3.6** (1.2-10.3)	**1.0** (0.4-2.5)	**1.7** (0.9-3.2)
**3-5 FSS**	**1.4** (1.0-2.0)	**1.6** (0.8-3.4)	**1.3** (0.6-2.8)	**1.8** (1.0-3.3)	**1.0** (0.5-2.2)	**0.6** (0.3-1.4)	**0.4** (0.1-2.9)	**1.3** (0.4-3.7)	**1.1** (0.3-4.0)	**0** (-)
**BDS**	**12.7** (11.0-14.6)	**15.6** (11.6-20.5)	**13.1** (9.8-17.4)	**15.6** (11.9-20.1)	**8.4** (6.1-11.5)	**7.9** (6.2-9.9)	**9.2** (5.2-15.9)	**12.8** (7.8-20.2)	**8.0** (5.3-12.1)	**4.7** (3.2-6.7)
**Single-organ BDS**	**9.8** (8.2-11.6)	**11.7** (8.3-16.2)	**9.4** (6.7-13.1)	**11.8** (8.5-16.1)	**7.2** (5.0-10.2)	**6.9** (5.3-8.8)	**8.4** (4.5-15.1)	**10.1** (5.7-17.2)	**7.2** (4.6-11.2)	**4.3** (2.9-6.3)
**CP subtype**	**1.3** (0.9-2.0)	**2.7** (1.4-4.9)	**1.4** (0.4-4.6)	**0.9** (0.4-2.1)	**0.9** (0.4-2.0)	**1.2** (0.6-2.5)	**1.6** (0.3-7.1)	**2.2** (0.5-9.0)	**1.2** (0.4-3.9)	**0.6** (0.2-1.7)
**GI subtype**	**4.9** (3.8-6.2)	**5.8** (3.9-8.7)	**3.7** (2.4-5.7)	**6.0** (3.6-9.8)	**4.2** (2.7-6.5)	**2.9** (2.1-4.0)	**3.2** (1.3-7.4)	**2.9** (1.1-7.3)	**3.4** (1.9-5.8)	**2.4** (1.4-3.9)
**MS subtype**	**3.6** (2.7-4.8)	**2.0** (1.0-4.1)	**4.4** (2.6-7.4)	**5.5** (3.3-9.1)	**2.4** (1.3-4.2)	**2.2** (1.4-3.5)	**1.7** (0.4-6.9)	**1.2** (0.4-3.8)	**4.1** (2.1-8.0)	**1.5** (0.8-2.9)
**GS subtype**	**3.7** (2.8-4.9)	**6.3** (3.7-10.4)	**4.2** (2.3-7.4)	**4.6** (2.8-7.3)	**1.4** (0.5-3.7)	**2.4** (1.5-3.9)	**2.9** (1.0-8.4)	**6.3** (2.8-13.5)	**1.8** (0.9-3.5)	**0.8** (0.3-1.8)
**Multi-organ BDS**	**3.0** (2.3-4.0)	**4.0** (2.4-6.6)	**3.8** (2.1-6.9)	**3.9** (2.4-6.5)	**1.3** (0.6-2.5)	**1.0** (0.5-1.9)	**0.8** (0.2-3.4)	**2.8** (1.0-7.8)	**0.8** (0.3-2.4)	**0.4** (0.1-1.4)

Abbreviations: FSS=Functional somatic syndrome; IB=irritable bowel; CWP=chronic widespread pain; CF=chronic fatigue; WAD=whiplash associated disorders; MCS=multiple chemical sensitivity; "X pure"=Only fulfilling criteria of that single FSS and no other FSS; BDS=bodily distress syndrome; CP=cardiopulmonary; GI=gastrointestinal; MS=musculoskeletal; GS=general symptoms type; CI=confidence interval

Table 2: Sex, social factors, physical health, and comorbidity of functional somatic syndromes

	No FSS (n=1222)	IB pure (n=70)	CWP pure (n=18)	CF pure (n=92)	WAD pure (n=10)	MCS pure (n=12)	IB (n=161)	CWP (n=96)	CF (n=228)	WAD (n=53)	MCS (n=33)	One FSS (n=202)	Two FSS (n=110)	≥ 3 FSS (n=45)
**Basic**, %														
Sex; Women	55 (52-58)	69 (56-79)	67 (41-87)	72 (61-81)	60 (26-88)	92 (62-100)	77 (70-83)	77 (67-85)	75 (69-81)	70 (56-82)	76 (58-89)	71 (64-77)	77 (68-85)	80 (65-90)
**Social**, %														
Cohabiting	77 (75-80)	64 (52-75)	82 (57-96)	76 (66-84)	70 (35-93)	67 (35-90)	65 (57-72)	71 (61-80)	69 (63-75)	70 (56-82)	64 (45-80)	72 (65-78)	67 (57-76)	64 (49-78)
Currently employed	65 (62-68)	72 (60-83)	53 (28-77)	70 (59-79)	70 (35-93)	67 (35-90)	58 (49-65)	43 (33-54)	60 (53-66)	55 (40-69)	53 (35-71)	69 (62-75)	53 (43-62)	40 (25-56)
Formerly employed	34 (31-36)	26 (16-38)	41 (18-67)	30 (21-41)	20 (3-56)	33 (10-65)	40 (32-48)	54 (43-64)	38 (31-44)	41 (28-56)	44 (26-62)	30 (23-36)	43 (33-52)	58 (42-73)
Have never been employed	1.3 (1-2)	1.4 (0.04-8)	6.0 (0.1-29)	0 (-)	10 (0.3-45)	0 (-)	3 (1-6)	3 (1-9)	3 (1-6)	4 (0.5-13)	3 (0.1-16)	2 (0.3-4)	5 (2-10)	2 (0.1-12)
No vocational training/0 years	11 (9-13)	19 (11-31)	6 (0.2-30)	16 (9-25)	40 (12-74)	8 (0.2-38)	19 (13-26)	14 (8-23)	17 (13-23)	15 (7-28)	16 (5-33)	17 (12-23)	18 (11-27)	16 (7-31)
Short vocational training /<3 years	18 (16-20)	9 (3-18)	25 (7-52)	19 (11-29)	10 (0.3-45)	17 (2-48)	15 (10-21)	19 (11-29)	18 (13-24)	10 (3-21)	13 (4-29)	15 (11-21)	18 (11-27)	16 (7-31)
Medium long vocational training /3-4 years	45 (42-48)	40 (28-53)	44 (20-70)	38 (28-49)	40 (12-74)	58 (28-85)	40 (32-48)	40 (30-51)	40 (33-47)	54 (39-68)	47 (29-65)	41 (34-48)	41 (31-51)	44 (29-60)
Long vocational training /> 4 years,	26 (24-29)	31 (21-44)	25 (7-52)	28 (19-38)	10 (0.3-45)	17 (2-48)	26 (20-34)	27 (18-37)	25 (19-31)	21 (11-35)	25 (11-43)	27 (21-34)	24 (16-33)	23 (12-39)
**Physical health**, %														
Poor self-perceived health[a]	17 (15-19)	21 (13-33)	35 (14-62)	45 (35-56)	30 (7-65)	42 (15-72)	37 (29-45)	60 (49-70)	50 (43-57)	53 (39-67)	45 (28-64)	35 (28-42)	47 (37-57)	67 (51-80)
Being limited in daily activities[b]	21 (19-23)	26 (16-38)	35 (14-62)	44 (34-55)	30 (7-65)	42 (15-72)	40 (32-48)	64 (53-74)	52 (45-59)	59 (44-72)	50 (32-68)	36 (29-43)	52 (42-62)	69 (53-82)
**GP diagnosis and comorbidity**, %														
Having FSS according to a doctor[c]	21 (19-23)	64 (51-75)	41 (18-67)	30 (21-40)	90 (56-100)	17 (2-48)	67 (59-74)	64 (53-73)	51 (45-58)	87 (75-95)	55 (36-72)	45 (38-52)	60 (50-69)	84 (71-94)
Physical comorbidity	57 (54-60)	76 (64-85)	72 (47-90)	68 (58-78)	90 (54-99)	92 (62-100)	74 (66-81)	76 (66-84)	71 (64-76)	83 (70-92)	82 (65-93)	74 (67-80)	71 (61-79)	80 (65-90)
Mental comorbidity	7 (6-9)	10 (4-20)	11 (1.4-35)	22 (14-32)	0 (-)	8 (0.2-39)	19 (13-26)	23 (15-33)	24 (19-30	8 (2-18)	12 (3-28)	15 (10-21)	23 (15-32)	24 (13-40)

Sex, social factors, physical health, and comorbidity of functional somatic syndromes (FSS); % (95% CI). No FSS=Participants not having any FSS; IB=irritable bowel; CWP=chronic widespread pain; CF=chronic fatigue; WAD=whiplash associated disorders; MCS=multiple chemical sensitivity; "X pure"=Only fulfilling criteria of that single FSS and no other FSS. GP = General practitioner. [a] Fair or poor health (41). [b]all of/most of/some of the time (41). [c]Received a diagnosis of at least one of the following: Irritable bowel syndrome, fibromyalgia, chronic fatigue syndrome, whiplash associated disorders, and multiple chemical sensitivity from a general practitioner

Table 3: Sex, social factors, physical health, and comorbidity of bodily distress syndrome

	No BDS (n=1177)	Single-organ BDS (n=327)	*CP subtype* (n=47)	*GI subtype* (n=173)	*MS subtype* (n=107)	*GS subtype* (n=111)	Multi-organ BDS (n=86)
**Basic**, %							
Sex; Women	55 (52-57)	70 (65-75)	68 (53-81)	69 (62-76)	74 (64-82)	72 (63-80)	83 (73-90)
**Social**, %							
Cohabiting	78 (76-81)	68 (63-73)	68 (53-81)	68 (61-75)	72 (62-80)	65 (55-74)	65 (54-75)
Currently employed	66 (63-69)	59 (53-64)	64 (49-77)	57 (50-65)	53 (43-63)	61 (52-70)	57 (45-67)
Formerly employed	33 (30-35)	39 (34-45)	34 (21-49)	42 (34-49)	44 (35-54)	37 (28-47)	39 (28-50)
Have never been employed	1.3 (1-2)	2 (1-4)	2 (0.1-11)	1 (0.1-4)	3 (1-8)	2 (0.2-6)	5 (1-12)
No vocational training/0 years	11 (9-13)	16 (12-20)	16 (7-30)	16 (11-22)	12 (6-19)	19 (12-27)	19 (11-29)
Short vocational training /<3 years	18 (16-20)	16 (13-21)	16 (7-30)	16 (11-22)	23 (16-33)	16 (9-24)	19 (11-29)
Medium long vocational training /3-4 years	45 (42-48)	42 (37-48)	41 (26-57)	40 (32-47)	45 (35-55)	41 (31-51)	36 (26-48)
Long vocational training/> 4 years	26 (24-29)	26 (21-31)	27 (15-43)	28 (22-36)	20 (13-30)	25 (17-34)	26 (17-37)
**Physical health**, %							
Poor self-perceived health[a]	17 (15-19)	36 (31-42)	32 (19-47)	31 (24-39)	45 (36-55)	44 (34-53)	56 (45-67)
Being limited in daily activities[b]	21 (18-23)	40 (35-46)	40 (26-56)	32 (25-40)	53 (43-63)	47 (38-57)	54 (42-65)
**GP diagnosis and comorbidity**, %							
Having FSS according to a doctor[c]	21 (19-23)	49 (43-55)	38 (24-53)	59 (51-66)	50 (41-60)	40 (31-50)	58 (47-69)
Physical comorbidity	56 (54-59)	74 (69-79)	70 (55-83)	76 (69-82)	72 (62-80)	70 (61-79)	70 (59-79)
Mental comorbidity	6 (5-8)	17 (14-22)	21 (11-36)	17 (12-24)	16 (10-24)	26 (18-35)	28 (19-39)

Sex, social factors, physical health, and comorbidity of bodily distress syndrome (BDS); % (95% CI). No BDS=Participants not having BDS; GP = General practitioner. [a] Fair or poor health (41). [b]all of/most of/some of the time (41). [c]Received a diagnosis of at least one of the following: Irritable bowel syndrome, fibromyalgia, chronic fatigue syndrome, whiplash associated disorders, and multiple chemical sensitivity from a general practitioner

Figure 1: Receiving Operating Characteristics curve of the bodily distress syndrome diagnosis

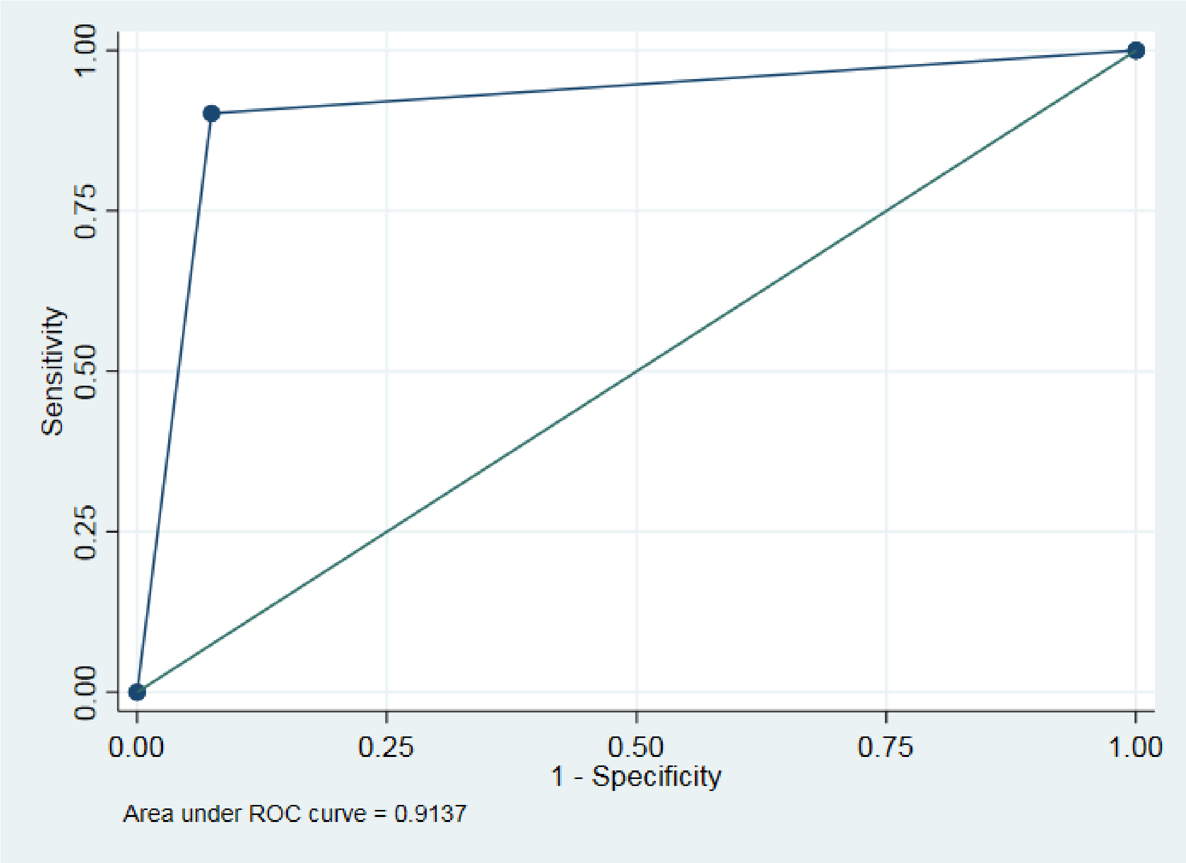

Receiving Operating Characteristics curve of the bodily distress syndrome diagnosis with respect to detecting a functional somatic syndrome diagnosis
